# Experiments upon the Superior Laryngeal Nerve

**Published:** 1890-01

**Authors:** L. Breisacher

**Affiliations:** Detroit, Michigan


### EXPERIMENTS UPON THE SUPERIOR LARYNGEAL NERVE.

By L. Breisacher, V.M.D.,
Detroit, Michigan.
(Centralblatt f. d. med. Wissenshaften.)

H. Möller, gave as the results of two experiments upon the superior laryngeal nerve of horses, that after section of this nerve before its entrance into the larynx, all the muscles of the larynx on the side upon which section of the nerve was made were found in a state of pronounced atrophy, and he concluded therefrom that in the superior laryngeal nerve trophic fibres were to be found. The horses had lived one-and-a-half and four-and-a-half months respectively. Following him laterly S. Exner, performed the similar experiment on a horse with excision of a piece of the nerve about five centimeters long. He found after one-and-a-half months a visible atrophy of the crico-arytenoid muscle on the side of the excision, and like Möller an atrophy with a yellow coloration of the muscles on the same side. He thinks the atrophy can be explained in the following way:—By the removal of the sensory nerves of the larynx disappears the impulses and resentments of movement, and hence the movement itself is diminished. The degenerations therefore found by Möller and himself occurring after excision of the superior laryngeal nerve of the horse are to be considered as atrophies of inactivity. I must remark that the information of Exner relative to the extension of the atrophy of the larynx muscles in Rabbits in Col. 1888, No. 24, and 1889, No. 6, differ.

The author cut a superior laryngeal nerve and resected a piece one-and-a-half inches long near its entrance to the thyroid cartilage, on horses which then lived three-and-a-quarter and three-and-three-quarter months respectively. At the autopsy it was proved each time that the nerve was resected in the intended manner, and the whole group of muscles on the operated as well as upon the side not operated on were found in an entirely normal state. Any atrophy of the group of muscles or yellow coloration of the same was not to be found. Neither has Dr. Breisaeher seen when making experiments upon dogs and rabbits after resection of the superior laryngeal nerve any degenerations of the laryngeal muscles supplied by the superior inferior nerve. Consequently there is no reason to suppose that there exist trophic fibres in the superior laryngeal nerve as Möller supposed, or atrophy of inactivity due to the want of impulses of movement, etc., as Exner, which would cause roaring in the horse.[1]

*Death of a Vienna Physician from Glanders.*—A most distressing case has occurred in the General Hospital in Vienna. In August, a man was brought to the hospital suffering from glanders, which he contracted from a horse. After death, Dr. Rowlaski, an expert in bacteriology made the autopsy, and produced pure cultures of the glanders bacillus from the virus. But a "Medical Thomas," Dr. Hoffman, expressed his doubts as to whether the bacillus had still in it, the power of infection. Dr. Rowlasks gave him one of his cultures to perform a series of experiments which proved affirmative. Early in October, having contracted a serious cold, which he treated by hypodermic injections, using the same syringe with which he had performed the experiment on the animals, Dr. Hoffman found himself seriously ill, and in a few days was covered with *tubercles* and ulcers, and died of acute glanders.

*Traumaitic Pericarditis.*—Dr. G. S. K. Hickman of Steelton, Pa., reports a case of this disease in which a wire, which had been the cause, had passed from the heart and was found at the autopsy imbedded in the left humeral artery.

*The action of Salt on Bacteria.*—Dr. Forster, Professor of Hygiene in the University of Amsterdam, has been investigating the action of common salt on pathogenic bacteria. and finds that the results are very different with different micro-organisms. The bacillus of cholera was very easily destroyed by the salt, even within a few hours, but on the other hand the tubercle and typhoid bacilli were not destroyed after weeks or even months of subjection to the action of the salt. These results, the author thinks, speak imperatively against the practice of salting down the flesh of tuberculous animals, with the idea that the pickling will render such meat safe in a short time.

*Dangers from Carbolic Acid.*—Prof. Billroth, of Vienna, gives a timely caution against the indiscriminate and unskilled use of carbolic acid: "I have lately seen four cases, in which fingers which had suffered a most insignificant injury became gangrenous through the uncalled-for application of carbolic acid. Carbolic acid is now much less used in surgery than formerly; we have only gradually become acquainted with its dangers. The acid may not only cause inflammation and gangrene, but also blood poisoning, and so may even prove fatal. It is useful only in the hands of a skillful surgeon, and ought never to be used without his advice. The best antidote in carbolic acid poisoning is soap, which should be taken immediately and repeatedly until all symptoms of poisoning disappeared." [Ed. note:—The same unlooked for result has occurred in our own practice, both in wounds on dogs and horses.]

*Eucalyptol as an Antiseptic.*—A thorough examination of the air of operation halls, in Vienna, which had been saturated with eucalyptol spray, showed that no development of bacteria cultures on gelatine takes place in them. According to these investigations, comments a contemporary, eucalyptol is to be regarded as an excellent disinfectant of the air, since it is harmless when inhaled, and does not destroy clothing or furniture.

